# Activation vs. Experiential Avoidance as a Transdiagnostic Condition of Emotional Distress: An Empirical Study

**DOI:** 10.3389/fpsyg.2018.01618

**Published:** 2018-09-03

**Authors:** Concepción Fernández-Rodríguez, Dolores Paz-Caballero, Sonia González-Fernández, Marino Pérez-Álvarez

**Affiliations:** Department of Psychology, University of Oviedo, Oviedo, Spain

**Keywords:** activation, experiential avoidance, emotional distress, anxiety, depression, transdiagnostic, contextual therapy

## Abstract

**Background:** From a contextual transdiagnostic approach, this study focuses on the importance of the processes of Experiential Avoidance and Activation in explaining and treating psychological problems. There exists widespread empirical evidence to suggest that the response pattern known as Experiential Avoidance, a general unwillingness to remain in contact with particular private experiences through the use of maladaptive avoidance strategies, acts as a functional dimension in various psychological problems. Activation, that is, maintaining contact with experiences/conditions of life and consequently with associated sources of reward, is a condition present in most therapeutic processes. Although a great deal of research has analyzed the relationship of the value of reward with the etiology and maintenance of psychological problems, Activation, as a transdiagnostic factor, has been studied less. The aim of this paper is to carry out an empirical study of the relationship between Activation, EA and emotional state and analyze the capacity of these two conditions to discriminate the intensity and symptomatology type in subjects with emotional distress.

**Methods:** The Hospital Anxiety and Depression Scale (HADS), Environmental Reward Observation Scale (EROS) and Behavioral Activation for Depression Scale (BADS) were completed by 240 health center users.

**Results:** Of the participants, 55% showed clinically relevant emotional distress. All cases of depression showed clinical anxiety. To discriminate between subjects without (*n* = 109) and with emotional distress (*n* = 131), analyses of the ROC curves and logistic regression analysis identified the BADS-Avoidance/Rumination followed by the EROS. To discriminate between subjects with anxiety but without depression (*n* = 61) and with anxiety and depression (*n* = 70), the most efficient scales were EROS followed by BADS-Social Impairment.

**Conclusion:** It was shown that people with no emotional complaints maintained greater contact with life experiences and with environmental sources of reward than those with emotional distress. Response patterns showing Experiential Avoidance and a reduction in Activation responses were associated with clinical distress. A reduction in Activation was the condition which distinguished those people with the greatest distress and also the greatest comorbidity of symptoms of depression and anxiety. These data support the transdiagnostic nature of Activation and suggest greater attention should be paid to this concept.

## Introduction

The current transdiagnostic and trans-therapy approach has arisen in response to the severe limitations affecting psychopathology and treatment approaches focusing on specific disorders (Mansell et al., 2009; Harvey et al., 2011; Sauer-Zavala et al., 2017). The main reasons for such limitations are well known. Firstly, after decades of research there exists abundant literature associating psychiatric disorders with a wide range of genes, biological markers and/or cerebral circuits. However, the mechanisms linked to each specific disorder remain unclear (Kendler et al., 1992; Caspi and Moffitt, 2006; Navarro-Mateu et al., 2013). Whereas etiological and epidemiological studies previously examined the contribution of specific genes and biomarkers toward explaining specific disorders in isolation, they now focus on comorbidity. That is, they examine the combined action of multiple factors – genetic and environmental – thought to be associated with the onset and maintenance of comorbid disorders (Cerdá et al., 2010; Kubota et al., 2012). Secondly, the validity of diagnostic systems (*Diagnostic and Statistical Manual of Mental Disorders*; American Psychiatric Association [APA], 2013) is compromised by high levels of comorbidity. The presence of simultaneous, multiple disorders is extremely common in the realm of psychopathology. Boschloo et al. (2015) used a network analysis in a data set with over 34000 patients interviewed on 120 symptoms of 12 major DSM-IV disorders. In the resulting network, no sharp boundaries were found between the 12 disorders. Within the different psychiatric disorders, comorbidity is particularly high amongst emotional disorders, with rates of between 50 and 95% depending on the study (Andrews et al., 2008; Al-Asadi et al., 2014). There do not appear to be differences between samples involving children and adolescents (Axelson and Birmaher, 2001; Johnco et al., 2015) and adult populations (Mackenzie et al., 2011). Thirdly, with regard to the treatment, there is abundant clinical evidence to suggest that comorbidity may remit during the treatment of a primary disorder (Newman et al., 2010); and, also, that specific treatments may also be useful for other disorders (Antony and Rowa, 2005). Regarding therapy approaches, there currently exists a broad range of psychological therapies which, in controlled studies, have proved efficient for a wide variety of psychological problems (American Psychological Association [APA], 2012). In this context, the search for alternative ways of explaining and addressing psychological problems has focused on those common factors believed to be at the root of how such problems are acquired and maintained and of the efficiency of the treatments.

There exists widespread empirical evidence to suggest that the response pattern known as Experiential Avoidance (EA) acts as a functional dimension in various psychological problems. Experiential avoidance is defined as the “phenomenon that occurs when a person is unwilling to remain in contact with particular private experiences (e.g., bodily sensations, emotions, thoughts, memories, behavioral predispositions) and takes steps to alter the form or frequency of these events and the contexts that occasion them” (Hayes et al., 1996, p. 1154). Although the avoidance of unwanted inner experiences may alleviate distress in the short-term, it paradoxically exacerbates distress over longer periods of time (Campbell-Sills et al., 2006; Bardeen, 2015). When this strategy becomes an inflexible pattern of behavior, the person’s functionality in different areas of life is affected. By avoiding experiences, people distance themselves from those conditions of life which are relevant to them, whilst at the same time losing contact with the very life contingencies/circumstances in which change could, and should, occur. Consequently, distress increases, and the person becomes trapped in a loop of avoidance (Hayes et al., 1999; Hayes et al., 2006; Kashdan et al., 2006).

Experiential avoidance has been hypothesized to play an important role in the etiology, maintenance and modification of various forms of psychopathology and has been suggested as a core vulnerability factor for emotional distress. Laboratory studies demonstrate that experiential avoidance amplifies anxiety symptomatology in individuals with no history of anxiety-related disorders (for reviews see: Chawla and Ostafin, 2007; Ruiz, 2010). In relation to depression, theoretical accounts suggest that EA alters an individual’s emotional experience, thereby increasing psychological distress and exacerbating depressive symptoms (Hayes et al., 2004). Studies involving adults converge on a strong positive relation between EA and depression, particularly among women with affective disorders (Spinhoven et al., 2014), although the relation between EA and depression is present even with sub-clinical levels of depression (Tull et al., 2004).

The opposite response to experiential avoidance is Acceptance-Activation. Acceptance can be defined as: “actively contacting psychological experiences -directly, fully, and without needless defense- while behaving effectively” (Hayes et al., 1996, p. 1157). Acceptance should not be regarded as a passive tolerance or a fatalistic resignation, but as the ability to embrace internal experiences (thoughts, emotions, etc.) as they occur. An acceptance approach does not abandon direct change efforts. It simply targets them toward more readily changeable domains, such as overt behavior or life situations, rather than personal history or automatic thoughts and feelings (Hayes et al., 1999). Contacting experiences in this way requires some kind of Activation, whether it be by affronting, despite the distress, those tasks/situations previously avoided, or by abandoning the struggle to control or suppress thoughts/emotions seen as threatening in order to focus on life situations. Activation releases the patient from the Experiential Avoidance trap through increases in goal- and value-based activity levels that elicit increased response-contingent reinforcement. To once again quote Hayes et al. (1996), it may indeed be that “many forms of psychopathology are not merely bad problems, they are also bad solutions” (p. 1162).

In contrast to the abundant literature regarding the transdiagnostic condition of EA, analyses of the relationship between Activation and emotional problems have been based principally on the effects of contextual therapies (Pérez-Álvarez, 2012). These therapies focus on accepting negative experiences rather than controlling them and on experiencing a greater contact with life situations. The efficacy of these interventions, shown in controlled studies, is presented as proof of the trans-therapy value of Activation (Kanter et al., 2010; Ekers et al., 2014; Zhang et al., 2018). As pointed out previously, Activation has also been recognized as an active ingredient in different transtherapeutic approaches (Meidlinger and Hope, 2017). As a transtherapeutic condition, Activation involves recovering positive environmental reinforcement. As a transdiagnostic condition, it stresses the role of environmental reinforcement in the onset and maintenance of psychological problems. The experience of environmental reward has been investigated by various disciplines within the biological and social sciences. Researchers have long since established a neurobiological basis of reward with a brain reward system considered a mediating factor in affective change (Gray, 1994; Corr, 2004), and analyzed the relationship of the value of the reward with the etiology and maintenance of psychiatric disorders (Gatzke-Kopp et al., 2009; Burkhouse et al., 2017). In other studies, inadequate environmental reward has consistently been highlighted as a mediator of negative affect (Lewinsohn, 1974; Martell et al., 2001; Hopko et al., 2003). The relationship between response-contingent positive reinforcement (RCPR) and emotional distress has been established (Cuijpers et al., 2007; Manos et al., 2010); in particular, a low level of RCPR is one of the critical predictors of clinical depression. Lewinsohn et al. (1980) explain the decrease in RCPR as a consequence of the combination of the following conditions: a decrease in the number of reinforcement events; a decrease in the availability of these reinforcers in the environment; the absence of appropriate instrumental behaviors to experience gratifying contingencies; and an increase in exposure to aversive environmental experiences. Increased behavioral activation and exposure to environmental reward appear to increase the positive affect (Carver, 2004; Gawrysiak et al., 2012).

In order to assess the relationship between RCPR and emotional state, it is vital to carry out an objective and valid evaluation of the former. In a therapy context, observation and behavioral registers are recommended as the ideal strategies for obtaining a direct measure of RCPR. However, this procedure is complicated, as it requires observation of relevant behaviors in the person’s day-to-day environment over a prolonged period. Manos et al. (2010) carried out an exhaustive critical review of the different strategies for measuring levels of positive reinforcement and stress, as measures of RCPR, the usefulness of such validated instruments as the Environmental Reward Observation Scale (EROS; Armento and Hopko, 2007); the Behavioral Activation for Depression Scale (BADS; Kanter et al., 2007), which also provides adequate measurement of Experiential Avoidance; or the Response Probability Index (RPI; Carvalho et al., 2011).

Activation, as the action which puts a person in contact with life experiences, can be considered an alternative response strategy to Experiential Avoidance. In this sense, Activation is simply the other face of EA, and we would be faced with a single transdiagnostic condition in which EA would be the response pattern involved in the development of psychological problems and Activation is proposed as the therapeutic strategy to alleviate the psychopathological effects of EA. There is evidence to suggest that focusing treatment on reducing EA and increasing Activation can lead to improvements in anxiety and emotional disorders (González-Fernández et al., 2018; Zhang et al., 2018). Acceptance and Commitment Therapy (ACT; Hayes et al., 1999), in particular, teaches clients to contact psychological experiences, directly and fully. Instead of abandoning change efforts completely, ACT targets them toward more readily changeable domains such as overt behavior or life situations, rather than thoughts and feelings.

Seen from a different perspective, it can be argued that Activation has, in its own right, transdiagnostic value. It is suggested that the presence or absence of Activation, that is, maintaining contact with experiences/conditions of life and consequently with associated sources of reward, could be said to explain, to some extent, the presence and intensity of manifestations of emotional distress. The transtherapeutic response to this transdiagnostic proposal would be to develop a treatment aimed at acting on the transdiagnostic process, that is, a treatment that promotes effective activation responses. The current intervention of Behavioral Activation (BA; Jacobson et al., 2001; Martell et al., 2001) is a therapy expressly directed at helping the subject to increase Activation in such a way that he/she can experience greater contact with the sources of reward and solve the problems in his/her life. A wide range of empirical studies are based on the use of BA in dealing with emotional problems in the general population (Lejuez et al., 2011; Doi et al., 2016) and in subjects with health problems (Fernández-Rodríguez et al., 2011, 2017; Bombardier et al., 2017).

From a transdiagnostic perspective, the aim of this study is to carry out empirical research into the relationship between Activation, EA and emotional state. In line with clinical evidence and previously mentioned studies, we expect to find, firstly, a high degree of comorbidity between anxiety and depression symptomatology, and secondly that the presence of patterns of Experiential Avoidance is related to emotional distress while maintaining contact with life experiences/conditions, with their subsequent reward, in other words Activation, is expected to be a response strategy which distinguishes subjects with no emotional complaints. On the other hand, the levels of Activation is expected to determine the intensity and type of manifestations of emotional distress. Ultimately, it is hoped that this study will offer new evidence regarding the transdiagnostic nature of Activation and Experiential Avoidance.

## Materials and Methods

### Recruitment and Procedure

A cross-sectional design was used (Ato et al., 2013). Over a 3-month period, a consecutive preselection of people visiting three Community Health Centres in urban areas to seek advice regarding assistance and/or health care was carried out. The following inclusion criterion was established: age between 16–75 years. The minimum age to take part in the study was established in accordance with the legal minimum age regarding health matters in Spain. The exclusion criterion was physical and/or cognitive deterioration which might hinder understanding and completing of measuring instruments. All those subjects who, during the established preselection period, fulfilled the criteria were invited to participate in the study. All were informed, verbally and in writing of the objectives and procedures of the study and of the guarantees regarding confidentiality in the evaluation and treatment of data. They were then asked to give written consent. None received economic remuneration or any other kind of compensation for taking part in the study. Each subject filled in the evaluation tests individually and without help. All the participants, in line with a written protocol, were given the same instructions. The evaluation room was suitable, in terms of facilities and privacy, to allow the tests to be carried out adequately. The tests took approximately 5–10 min.

The initial sample was of 278 people, of which 36 (14.8%) refused to participate. The final sample consisted of 242 people, 214 women and 28 men, aged between 16 and 73 years of age (*M* = 49.60; *SD* = 10.25). Regarding marital status, 15.3% were single, 71.8% married or living with a partner, 11.8% were separated/divorced and the rest widows/widowers. The majority of the participants lived with other family members, 5% lived alone. Regarding level of studies, 26.5% had basic studies, 38.5% secondary education and 35% had received university education. Regarding work situation, 49.5% were in work, 32.5% were unemployed and 18% retired.

### Measures

#### Hospital Anxiety and Depression Scale (HADS; Zigmond and Snaith, 1983)

The HADS is a 14-item scale with 2 subscales: Anxiety (HADS-A) and Depression (HADS-D), that was originally designed to detect emotional distress in non-psychiatric patients treated at hospital clinics. The total HADS score ranges from 0 to 42 while the subscales range from 0 to 21. Most of the items of the HAD-D concerning depression measure cognitive and emotional aspects. The anxiety items are also focused on the cognitive and emotional aspects of anxiety. In the depression and anxiety subscales, scores of 8–10 indicate probable cases and scores over 10 indicate clinical cases. For anxiety (HADS-A), this gave a specificity of 0.78 and a sensitivity of 0.90. For depression (HADS-D), this gave a specificity of 0.79 and a sensitivity of 0.83. (Smarr and Keefer, 2011). Compared to commonly used depression and anxiety measures, such as Beck Depression Inventory (BDI-II; Beck et al., 1996), The Center for Epidemiologic Studies Depression Scale (CESD; Radloff, 1977), The State-Trait Anxiety Inventory (STAI; Spielberger et al., 1983), or The Zung Self-Rating Depression Scale (Zung, 1965), correlations with the HADS-D and HADS-A ranged between 0.60 (good) and 0.80 (very good). Cronbach’s alpha for HADS-A varied from 0.68 to 0.93 (mean 0.83) and for HADS-D from 0.67 to 0.90 (mean 0.82) (Bjelland et al., 2002; Smarr and Keefer, 2011).

#### Environmental Reward Observation Scale (EROS; Armento and Hopko, 2007)

The EROS is a 10-item self-administered questionnaire answered using a 4-option Likert, which supplies information regarding the quantity and availability of reinforcement received from the patient’s environment. It was designed to evaluate the RCPR according to the formulation of Lewinsohn (1974). The items evaluate both the number of events that are potentially reinforcing and the availability of reinforcement in the environment (e.g., “A lot of activities in my life are pleasurable,” “Other people seem to have more fulfilling lives,” “Activities that used to be pleasurable are no longer gratifying,” “My life is boring”) and the competence of the subject to obtain reinforcement from the environment (e.g., “It is easy for me to find enjoyment in my life,” “I wish that I could find more hobbies that would bring me a sense of pleasure,” “I am satisfied with my accomplishments,” “The activities I engage in usually have positive consequences”). In its original version, with a sample of American students, the scale showed a unifactorial structure with high internal consistency (*α* = 0.85) and correlations, from moderate to strong, with different depression scales BDI-II = -0.78; CESD = -0.79; Zung Self-Rating Depression Scale = -0.54. In other validation studies which included the general population and also populations with emotional disorders, carried out with participants who were Spanish (Barraca and Pérez-Álvarez, 2010), Colombian (Valderrama-Díaz et al., 2016), or Belgian (Wagener and Blairy, 2015), in all cases there were found to be appropriate levels of internal consistency and also significant signs of construct validity. Here the Spanish adaptation of the EROS was used (Barraca and Pérez-Álvarez, 2010), for which data is available confirming its reliability (*α* = 0.86) and validity (correlations with the BDI-II = -0.73; STAI-E = -0.80; STAI-R = -0.70; BADS-T = -0.69).

#### Behavioral Activation for Depression Scale (BADS; Kanter et al., 2007)

The BADS consists of 25 items using a 7-point Likert scale measuring four dimensions: Activation, Avoidance/Rumination, Work/School Impairment and Social Impairment. The scale provides scores for each of the dimensions and a total score. The first subscale of the BADS, Activation, consists of seven items that assess goal-directed activation and demonstrated high internal consistency (e.g., “I did something that was hard to do but it was worth it,” “I engaged in a wide and diverse array of activities,” “I am content with the amount and types of things I did”). Second, the Avoidance/Rumination subscale consists of eight items that demonstrated high internal consistency. Avoidance as measured by this subscale focuses on avoidance of aversive thoughts and feelings (e.g.,“My activities were motivated by a desire to feel good rather than a desire to accomplish a goal,” “Most of what I did was to escape from or avoid something unpleasant,” “I avoided others who might feel badly and bring me down,” “I kept trying to think of ways to solve a problem but never tried any of the solutions”). The high correlation of this subscale with Acceptance and Action Questionnaire-II (AAQ-II, Bond et al., 2011) suggests that it focuses on the notion of Experiential Avoidance (Hayes et al., 1996). The final two subscales, the Work/School Impairment (e.g., “I took time off of work/school simply because I was too tired or didn’t feel like going in,” “I stayed in bed for too long even though I had things to do”) and the Social Impairment (e.g., “I was not social, even though I had opportunities to be,” “I did things to cut myself off from other people”), each contain five items and both evidenced acceptable internal consistency. The emphasis is placed on behaviors that are directed toward the accomplishment of goals that the individual has determined to be important in each area. High scores in Activation and in the total score show a higher level of Activation, whilst higher scores in the other dimensions indicate greater avoidance patterns. The Spanish adaptation of the BADS (Barraca et al., 2011) proved to be valid (significant correlations with the BDI-II = -0.63; STAI-E = -0.68; STAI-T = -0.70; EROS = 0.69) and had internal consistency (between *α* = 0.76 and *α* = 0.90). Factor analysis confirmed the four dimensions of the original instrument.

### Statistical Analysis

Since the assumption of normality was not fulfilled in some of the variables studied, the analysis of the relationships between the presence and degree of emotional distress and the response patterns of Activation and Experiential Avoidance was carried out using non-parametric tests. To analyze the capacity of the EROS and of the subscales of the BADS to distinguish between subjects without vs. with clinically relevant emotional distress, and, within the latter, between subjects with anxiety but without depression vs. those with anxiety and depression, *ROC* curves were used. Binary logistic regression analyses were also carried out to determine which combination of variables best discriminated between the groups.

In order to compare groups regarding sociodemographic variables, the chi-square test was used for the categorical variables (sex, marital status, level of studies, work situation) and the Kruskal–Wallis test for quantitative variables with important asymmetry (age, number of people in the household).

Analyses were all carried out using the SPSS-22 statistical package.

## Results

### Randomization Sample

The cut-off points on the Anxiety and Depression subscales of the HADS showed that 45% (*n* = 109) of the participants were not suffering from clinically relevant emotional distress (HADS-A*_M_*_= 4.28;_ *_SD_*_= 1.93_; HADS-D*_M_*_= 2.00;_ *_SD_*_= 1.95_). Of those participants with emotional distress (*n* = 133), 61 reached scores indicating probable/clinical cases of anxiety (HADS-A*_M_*_= 10.93;_ *_SD_*_= 2.33_; HADS-D*_M_*_= 4.62;_ *_SD_*_= 1.98_), 70 probable/clinical cases of anxiety together with depression (HADS-A*_M_*_= 13.49;_ *_SD_*_= 2.99_; HADS-D*_M_*_= 11.26;_ *_SD_*_= 3.08_), and only 2 probable cases of depression alone (HADS-D*_M_*_= 8;_ *_SD_*_= 0;_ HADS-A*_M_*_= 5;_ *_SD_*_= 0_). Subsequently, in line with the objectives of the study, the total sample was divided into three groups: (a) subjects without clinically relevant emotional distress; (b) subjects with anxiety but without depression; and (c) subjects with anxiety and depression. The only two people with scores in the HADS-A < 8 and in the HADS-D = 8 were excluded from the analysis. The final sample comprised 240 subjects. In none of the sociodemographic conditions were statistically significant differences found between the groups subsequently established according to the emotional state of the participants (see **Table 1**).

**Table 1:** Data regarding sociodemographic variables by groups and results of statistical tests used to compare them.

		Without clinical	Anxiety but without	Anxiety and	*χ^2^*	*df*	*p*
		emotional distress	Depression	Depression			
		(*n* = 109)	(*n* = 61)	(*n* = 70)			
Age	*M*(*SD*)	50.906 (9.197)	48.283 (11.665)	47.691 (9.591)	5.832ˆ*	2	0.056
Sex *(%)*	Male	9.2	9.8	17.1	2.892	2	0.235
	Female	90.8	90.2	82.9			
Marital status *(%)*	Single	15.3	13.5	18.2	0.650	6	0.996
	Married/	71.8	73.1	69.7			
	living with a partner						
	Separated/divorced	11.8	11.5	10.6			
	Widowed	1.2	1.9	1.5			
Level of studies *(%)*	Illiteracy	1.2		1.6	2.369	6	0.883
	Primary school	28.0	21.3	26.2			
	Secondary school	40.2	42.6	34.4			
	University	30.5	36.2	37.7			
Work situation *(%)*	In work	53.6	44.9	49.2	1.209	4	0.877
	Unemployed	29.8	36.7	30.8			
	Retired	16.7	18.4	20.0			
Number of people in	*M*(*SD*)	1.937 (1.158)	1.958 (1.129)	2.297 (.954)	4.735ˆ*	2	0.094
household *(%)*							

*^∗^The Kruskal–Wallis test was used; df, degrees of freedom.*

### Preliminary Analysis

The mean scores for the subjects without clinically relevant emotional distress, subjects with anxiety but without depression, subjects with anxiety and depression in the EROS and in the 4 subscales of the BADS are shown in **Table 2**.

**Table 2:** Descriptive statistics used in the EROS and in the scales of the BADS.

	Without clinically		Anxiety but without		Anxiety and depression	
	emotional Distress		Depression			
	*M*	*SD*	*M*	*SD*	*M*	*SD*
EROS	32.94	5.18	28.41	5.49	20.90	4.69
BADS-A	27.62	9.18	24.06	8.26	17.64	8.79
BADS-WI	4.45	4.93	8.86	6.64	16.71	7.63
BADS-SI	2.21	4.01	4.98	6.48	15.26	7.98
BADS-A/R	9.48	8.38	19.95	10.13	29.57	9.40

*EROS, Environmental Reward Observation Scale; BADS-A, Behavioral Activation for Depression, Scale-Activation; BADS-WI, Behavioral Activation for Depression, Scale-Work impairment; BADS-SI, Behavioral Activation for Depression, Scale-Social impairment; BADS-A/R, Behavioral Activation for Depression, Scale-Avoidance/Rumination.*

The correlations amongst the variables of the study are shown in **Table 3**. The results show significant relationships between the scores for anxiety, depression and the response patterns of activation and experiential avoidance.

**Table 3:** Correlations between study variables.

Variables	1	2	3	4	5	6	7
(1) HADS-A	–						
(2) HADS-D	0.767ˆ**	–					
(3) EROS	–0.691ˆ**	–0.784ˆ**	–				
(4) BADS-A	–0.397ˆ**	–0.505ˆ**	0.534ˆ**	–			
(5) BADS-WI	0.564ˆ**	0.677ˆ**	–0.667ˆ**	–0.444ˆ**	–		
(6) BADS-SI	0.580ˆ**	0.705ˆ**	–0.636ˆ**	–0.374ˆ**	0.622ˆ**	–	
(7) BADS-A/R	0.684ˆ**	0.673ˆ**	–0.654ˆ**	–0.321ˆ**	0.625ˆ**	0.629ˆ**	–

*(1) HADS-A, Hospital Anxiety and Depression Scale, Anxiety subscale; (2) HADS-D, Hospital Anxiety and Depression Scale, Depression subscale; (3) EROS, Environmental Reward Observation Scale; (4) BADS-A, Behavioral Activation for Depression, Scale-Activation; (5) BADS-WI, Behavioral Activation for Depression, Scale-Work impairment; (6) BADS-SI, Behavioral Activation for Depression, Scale-Social impairment; (7) BADS-A/R, Behavioral Activation for Depression, Scale-Avoidance/Rumination; ^∗∗^*p* < 0.01 (bilateral).*

### Variables Permitting Discrimination Between Subjects With vs. Subjects Without Emotional Distress

**Table 4** shows the results of the *ROC* analyses to evaluate the efficacy of the EROS and the 4 subscales of the BADS in distinguishing between subjects without vs. with emotional distress. The diagnostic efficacy of each of the scales was evaluated according to the area under the curve (*AUC*). The largest *AUC* corresponded to the score in the Avoidance/Rumination subscale of the BADS (*AUC* = 0.868, *p* < 0.001) followed by the total score in the EROS (*AUC* = 0.850, *p* < 0.001).

**Table 4:** Results of the analysis of the *ROC* curves to distinguish between subjects without emotional distress (*n* = 109) and with emotional distress (*n* = 131).

	EROS	BADS-A	BADS-WI	BADS-SI	BADS-A/R
*AUC*	0.850	0.712	0.814	0.791	0.868
*S.E.*	0.025	0.034	0.027	0.029	0.023
*Sig.*	<0.001	<0.001	<0.001	<0.001	<0.001

*AUC, area under the curve; S.E., standard error; EROS, Environmental Reward Observation Scale; BADS-A, Behavioral Activation for Depression, Scale-Activation; BADS-WI, Behavioral Activation for Depression, Scale-Work impairment; BADS-SI, Behavioral Activation for Depression, Scale-Social impairment; BADS-A/R, Behavioral Activation for Depression, Scale-Avoidance/Rumination.*

The five variables above were used as covariables in a binary logistic regression analysis to determine which combination of variables best discriminated between the two groups. Using the Forward-Conditional variable selection method, the variables included in the model were BADS-Avoidance/Rumination and EROS, in that order (see **Table 5**). The Nagelkerke R squared value was 0.499 for the Avoidance/Rumination subscale of the BADS and 0.568 when the score in the EROS was included together with the first variable. The percentage of correct classifications was 80.4% (25.9% above the random score), giving a sensitivity of 0.820 and a specificity of 0.785.

**Table 5:** Results of the logistic regression analysis to discriminate between subjects without vs. with emotional distress based on the EROS and the four subscales of the BADS.

		*B*	*S.E.*	*Wald*	*df*	*Sig.*	*Exp(B)*
Step 1	BADS-A/R	0.150	0.019	62.934	1	<0.001	1.162
	*Constant*	–2.299	0.335	47.103	1	<0.001	0.100
Step 2	EROS	–0.138	0.034	16.949	1	<0.001	0.871
	BADS-A/R	0.107	0.021	25.016	1	<0.001	1.112
	*Constant*	2.400	1.168	4.223	1	<0.040	11.027

*df, degrees of freedom; BADS-A/R, Behavioral Activation for Depression, Scale-Avoidance/Rumination; EROS, Environmental Reward Observation Scale.*

### Variables Permitting Discrimination Between Subjects With Anxiety vs. With Anxiety and Depression

The results of the *ROC* analysis to determine the capacity of the EROS and of the four subscales of the BADS to discriminate between subjects with anxiety but without depression and subjects with anxiety and depression are shown in **Table 6**. The greatest *AUC* was that corresponding to the total score in the EROS followed by the Social Impairment scale of the BADS.

**Table 6:** Results of analysis of *ROC* curves to discriminate between subjects with anxiety but without depression (*n* = 61) and subjects with anxiety and depression (*n* = 70).

	EROS	BADS-A	BADS-WI	BADS-SI	BADS-A/R
*AUC*	0.851	0.703	0.772	0.837	0.757
*S.E.*	0.034	0.046	0.042	0.036	0.043
*Sig.*	<0.001	<0.001	<0.001	<0.001	<0.001

*AUC, area under the curve; *S.E*., standard error; EROS, Environmental Reward Observation Scale; BADS-A, Behavioral Activation for Depression, Scale-Activation; BADS-WI, Behavioral Activation for Depression, Scale-Work impairment; BADS-SI, Behavioral Activation for Depression, Scale-Social impairment; BADS-A/R, Behavioral Activation for Depression, Scale-Avoidance/Rumination.*

As in the previous case, a binary logistic regression analysis was carried out to determine which combination of variables best discriminated between the groups. The variables included in the model were EROS and BADS-SI in that order (see **Table 7**). The value of *Nagelkerke’s R squared* was 0.463 when the first variable was included and 0.561 when both were considered. The percentage of correct classifications was 76.6% (22.7% above the random score), with a sensitivity of 0.812 and a specificity of 0.746.

**Table 7:** Results of the logistic regression analysis to discriminate between subjects with anxiety but without depression and subjects with both anxiety and depression using the EROS and the four BADS subscales.

		*B*	*S.E.*	*Wald*	*df*	*Sig.*	*Exp(B)*
Step 1	EROS	–0.295	0.054	30.033	1	<0.001	0.745
	*Constant*	7.360	1.335	30.395	1	<0.001	1571.655
Step 2	EROS	–0.216	0.055	15.428	1	<0.001	0.806
	BADS-SI	0.124	0.035	12.758	1	<0.001	1.132
	*Constant*	4.252	1.458	8.508	1	<0.004	70.249

*EROS, Environmental Reward Observation Scale; BADS-SI, Behavioral Activation for Depression, Scale-Social impairment.*

## Discussion

In line with the contextual transdiagnostic construct, the data indicated a high level of comorbidity amongst patients with a symptomatology of depression and anxiety. As predicted, Activation showed an inverse relationship with emotional distress while Experiential Avoidance showed a positive one.

More than half of the participants in the study showed a symptomatology compatible with a probable/clinical case of both anxiety and depression or only anxiety. This percentage may appear rather high. The prevalence of emotional disorders among general practice patients has been calculated at between 12 and 25% (Gates et al., 2016; Harrison et al., 2017). The heterogeneity of the percentages may largely be due to the diagnostic classification criteria, the instruments used or the country where the study is carried out. Using a Spanish population, other studies have also found higher levels, of between 20 and 50% (Sicras-Mainar et al., 2007). Be the figures as they may, researchers commonly conclude that emotional disorders amongst health service patients tend to be underestimated. Gates et al. (2016) found that less than 4% of patients in family medicine were diagnosed with depression or anxiety and concluded that such rates were significantly below the prevalence reported in the literature across primary care disciplines. The high percentage of women in the study sample could be another reason for the high rate of emotional distress observed. Emotional problems are known to be more prevalent amongst women (Kuehner, 2017). Furthermore, for reasons of a cultural nature, women are more willing than men to acknowledge the presence of diseases and seek attention for both their own health problems and those of people close to them (Gil-Lacruz and Gil-Lacruz, 2010). These facts, together with the guarantees offered by the participant selection procedure employed -a random selection in different health centers over a period of time- support the representativeness of the study sample and the generalization of the results to reflect the population studied.

Despite the clinical and sociodemographic importance of the high prevalence of emotional distress detected amongst users of community health services, one of the objectives of this study was to show the high levels of comorbidity. Such levels, which compromise the validity and usefulness of categorical diagnostic systems and psychological treatments focusing on specific disorders, have been found in numerous other studies (Boschloo et al., 2016). In the sample of mainly female community health service users with medium-high levels of emotional distress, all the probable/clinical cases of depression were also of anxiety. Only two people showed, exclusively and specifically, symptomatology indicating a probable clinical case of depression. These were excluded from the study sample due to the unviability of any statistical analysis of such a small group. Specific cases of anxiety were, however, identified, this condition affecting less than half of those suffering from clinical emotional distress. In the rest of cases, the comorbidity between symptoms of depression and anxiety was total. Given this situation and assuming the validity of the diagnostic measure used, it is possible to affirm that, in the study sample, anxiety and depression are not identified as specific and differentiated psychological problems.

The HADS (Zigmond and Snaith, 1983) was chosen to evaluate emotional distress as this scale excludes, to a large degree, the somatic element which could give false positives for anxiety or depression and which could well occur given that subjects were selected amongst people attending health centers/services. Furthermore, the Spanish version of the HADS has been shown to be a reliable, sensitive and valid tool for the screening of psychiatric morbidity, especially mood and anxiety disorders, in general hospital outpatients (Terol et al., 2007). There do, however, exist certain discrepancies regarding the clinical case cut-off points. Other validation studies for the scale using populations of Spanish health-service users have suggested lowering the cut-off points for anxiety and depression (Herrero et al., 2003). For this reason, in this study, it was thought appropriate to include probable cases of anxiety or depression as positive cases of emotional distress.

The emotional distress of the participants can be described in a dimension of increasing intensity. This situation is, by definition, predictable regarding people without vs. with an emotional distress. What is, however, noteworthy is the fact that, among the clinical cases, the group of anxiety without depression showed the lowest average values of emotional distress with lower average scores in the HADS subscales; while it is the group with both depression and anxiety which showed the greatest distress, as showed by its higher average scores both in the anxiety and depression scales of the HADS, which always reach values indicative of clinical cases. The participants’ Activation and Experiential Avoidance patterns can similarly be described in a range of increasing intensity. As the emotional distress of the participants increases, the Activation responses diminish, and the Experiential Avoidance responses increase. The group of subjects with symptomatology compatible with clinical cases of anxiety offers less Activation responses and more EA patterns than the group of subjects without emotional distress, while at the same time showing a higher degree of Activation and a lower degree of EA than the group of subjects with symptomatology compatible with clinical cases of both depression and anxiety. Indeed, it is the subjects showing clinical case symptomatology of depression and anxiety that report the lowest levels of Activation and the greatest increase in EA. Similarly, as mentioned previously, it is these same subjects that show the greatest intensity of emotional distress in the HADS. Consequently, it could be said that the different levels of emotional distress reported by the participants in the study are related to a lower number of activities aimed at achieving specific goals (work and social), a loss of environmental reinforcement and patterns of avoidance negative thoughts and feelings. These results are congruous with the contextual transdiagnostic approach, which aims precisely to discover, in the patterns of the subjects’ responses, the conditions which explain the onset and maintenance of psychological problems.

Due to the high comorbidity between their anxiety and depression symptomatology it was impossible to differentiate between the sample with regard to a single, specific psychopathological diagnostic category. Differentiation was, however, possible using the patterns of Activation and EA shown. The scores in the subscale Avoidance/Rumination of the BADS, followed by the total score in the EROS, made it possible to discriminate, with a high degree of sensitivity and specificity, between the subjects without vs. with clinical cases anxiety and/or depression. Consequently, it can be concluded that the avoidance of adverse thoughts and feelings and the loss of reinforcement in the environment appear to be the two conditions with the greatest capacity to explain the presence of emotional problems. Rumination, as a strategy to avoid adverse situations/experiences, has been clearly and repeatedly associated with the onset and subsequent development of depression (Spinhoven et al., 2016; Venta et al., 2017). Like any form of avoidance, it can lead to greater social isolation and, consequently, to reduced exposure to environmental sources of reward (Ferster, 1973). Furthermore, it is a strategy associated with the absence of attempts to actively solve problems, which also leads to reduced social participation (Martell et al., 2001). Rumination aimed at avoiding bodily perturbation also plays a part in the onset and maintenance of anxiety (Barlow et al., 2004; Abramowitz and Moore, 2007). In short, the avoidance of thoughts and feeling due to rumination leads, in varying degrees, to a loss of functionality in different areas of a person’s life and, consequently, to a loss of environmental reward. The existence of this effect appears to be supported by the sensitivity and specificity shown in the EROS scores, together with the BADS-A/R to discriminate between participants in this study without vs. with depression and/or anxiety. Our results are, therefore, congruent with those of many other studies which show that EA plays an important role in the etiology, maintenance and modification of depression and anxiety (Chawla and Ostafin, 2007; Ruiz, 2010).

It was, however, a reduction in Activation that was identified as the principal condition for distinguishing between the intensity and different manifestations of emotional distress. The results showed how the total score in the EROS and the Social Impairment subscale of the BADS discriminated satisfactorily between those subjects with anxiety and those with anxiety and also depression. The decrease in social functionality which appeared to be a differentiating condition of those subjects with more severe emotional distress could not be attributed to any specific sociodemographic or clinical condition, at least in the group evaluated in this study. Previous data suggest that this decrease in social functionality is due to the progressive reduction in participation in everyday social situations, this, in turn, being the result, principally, of Experiential Avoidance. There could, however, also be other reasons, such as a loss of sources of reinforcement in the social environment and/or the presence of particular life situations for which the person lacks efficient response strategies, or even because the actions of those close to the person in fact facilitate and reinforce the avoidance of those conflictive situations (Lewinsohn et al., 1980). Whatever the reason, the more a person loses contact with their environment, the less chances they will have of developing alternative responses (Lewinsohn, 1974; Martell et al., 2001; Hopko et al., 2003). Activation appears to be closely related to Experiential Avoidance. The following question then arises: Are these simply two faces of the same transdiagnostic condition (EA) or, as has been suggested, could the levels of activity which ensure the greatest environmental reinforcement contribute to explaining and discriminating between the different forms of emotional distress?

In this study, the presence of EA together with a reduction in Activation responses proved to be the combination which best discriminated between people without vs. with emotional distress. This result can undoubtedly be seen as yet further evidence of the role played by EA in psychological problems, especially bearing in mind that loss of functionality could well be as a result of the EA (Hayes et al., 2012). In this sense, there appears to be little justification for seeing Activation as a transdiagnostic condition that is independent of EA. On the contrary, Activation and Experiential Avoidance appear to be two different faces of the same transdiagnostic condition. However, our data also showed that, in terms of discriminating between the intensity and type of manifestations of emotional distress, the relative importance of Activation is greater. Indeed, it was a reduction in Activation responses combined with a loss of social functionality that best discriminated between those subjects with only anxiety symptomatology and those with both anxiety and depressive symptomatology, the latter being the group which showed the greatest degree of distress. If, as previously stated, the interaction between a reduction in Activation and an increase in EA appeared to play an important part in emotional distress (González-Fernández et al., 2017), these results also suggest that it could be a reduction in Activation which, on its own, contributes to the consolidation of different psychological problems. Indeed, strong evidence in favor of this idea is to be found in the therapeutic benefits of focusing treatment of emotional disorders on increasing Activation (Ekers et al., 2014; Meidlinger and Hope, 2017). In view of this, Activation would be of interest not only for its trans-therapy value, but also for its potential transdiagnostic value. It is not merely a question of showing that a reduction/loss of Activation exacerbates psychological problems. The process of unworkable action and its role in psychological inflexibility (Hayes et al., 2006) has already done that. The question is, rather, to what extent Activation, as a relevant condition in its own right, is capable of explaining a psychological problem?

Testing this hypothesis will clearly require further studies which reach beyond the limitations of this one. With regard to the instruments used to measure the variables in the study, it would be of particular interest to use different measures when evaluating the pattern of EA (e.g., AAQ-II), and for evaluating emotional problems (e.g., BDI-II, CESD, STAI) and similarly, to use samples which include different psychological disorders as well as being appropriately representative of the population being studied. The sample used in this study contains a high percentage of women. The fact that it is more frequent for women to suffer from and/or consult a doctor about emotional distress has been associated, amongst other things with biological vulnerability, thought patterns, and emotion reactivity and regulation (Nolen-Hoeksema and Jackson, 2001; Hyde et al., 2008; Spinhoven et al., 2016). It is, therefore, conceivable that the degree of distress observed in this study might be different men and women were more equally represented in the sample. If that were so, the strength of the relationships observed between the variables of this study might also be different. Generalization of data also depends on how representative the sample is and in the case of our study, the sample could only be considered representative of a population of community health center users in an urban area, more precisely, a medium-sized city, principally dedicated to the administrative and service sector. Consequently, in future studies it would be useful to employ samples which were suitably balanced with regard to gender and socioeconomic status in order to permit greater scope for generalization to the general population. Longitudinal designs could also be useful in providing reliable information regarding effects of Activation and Experiential Avoidance on the onset and maintenance of psychological problems. Other useful designs would be ones which, by analyzing each individual case, explain what aspects of the person’s interaction with his/her socio-cultural context maintain and weaken Activation responses. As stated by Meidlinger and Hope (2017), given that the evaluation that guides treatment is based mostly on functional analysis rather than symptoms or construction measures, functional analysis is a suitable method to move toward a new and deeper understanding of psychopathology.

## Conclusion

The results of the study show the high degree of comorbidity between depression and anxiety and support the need for a transdiagnostic approach. It was shown that people with no emotional complaints maintained greater contact with life experiences/conditions and with environmental sources of reward than those with emotional distress. Response patterns showing Experiential Avoidance and a reduction in Activation responses were associated with clinical distress. Furthermore, a reduction in Activation was the condition which distinguished those people with the greatest distress and also the greatest comorbidity of symptoms of depression and anxiety. As a first step, these findings suggest that more attention should be paid to Activation as a transdiagnostic condition involved in the explanation for different manifestations of emotional distress.

## Ethics Statement

The study was conducted in accordance with codes of ethics and conduct specified by the American Psychologist Association. All subjects gave written informed consent in accordance with the Declaration of Helsinki. The protocol was approved by the Research Ethics Committee of the Principality of Asturias, Spain (Ref.: 45/14).

## Author Contributions

CF-R has directly participated in the planning, execution and analysis of this study and has written the final version submitted. DP-C has directly participated in the planning and analysis of this study and has approved the final version submitted. SG-F has directly participated in the planning, execution and analysis of this study and has approved the final version submitted. MP-A has directly participated in the planning of this study and has approved the final version submitted.

## Conflict of Interest Statement

The authors declare that the research was conducted in the absence of any commercial or financial relationships that could be construed as a potential conflict of interest.
